# Supplementary material for: How to Overcome a Snail? Identification of Putative Neurotoxins of Snail-Feeding Firefly Larvae (Coleoptera: Lampyridae, Lampyris noctiluca)
Source: Toxins (Basel). 2024 Jun 14;16(6):272. doi: 10.3390/toxins16060272 (PMC11209139; doi:10.3390/toxins16060272)
Supplement: Supplementary file 1 [file toxins-16-00272-s001.zip › Supplementary Materials.pdf]

**Supplementary Material S1.** Table of all midgut compounds identified in *L. noctiluca* by a combined transcriptomic and proteomic approach.

**Supplementary Material S2**. Reproducibility of MALDI TOF mass spectra (m/z 2000 and 4500) of midgut secretions from *L. noctiluca.* Secretions were extracted repeatedly from four specimens. (A) Mass spectra that were reproduced for ¾ of the used specimens. Signals occurring in at least half of the 13 generated spectra, are highlighted in red. (B) Examples of deviating mass spectra compared to one of the reproducible mass spectra of specimen 1. The deviating spectrum from specimen 3 was obtained directly after collection and the deviating signals, likely are the result of recent food intake. Specimen 4 showed several additional signals, though many of the reproducible signals were still present with low intensity (not visible in the shown spectrum).

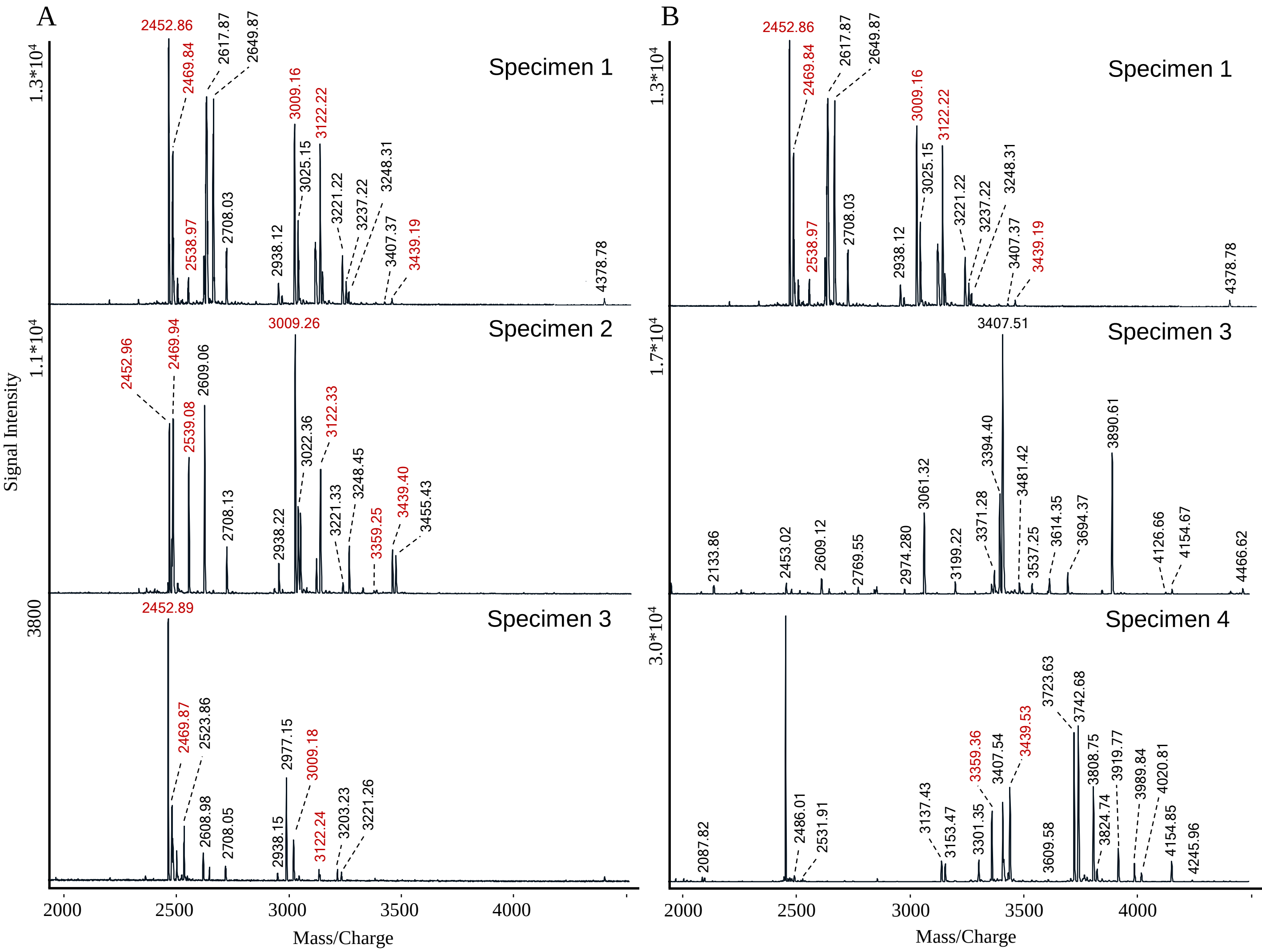

**Supplementary Material S3**. Prediction of three-dimensional structure for midgut compounds identified for *L. noctiluca with* Alphafold 2. (A, B) Threedimensional depiction of U-Lampyristoxin-Ln1a (C, D) Three-dimensional depiction of U-Lampyristoxin 2

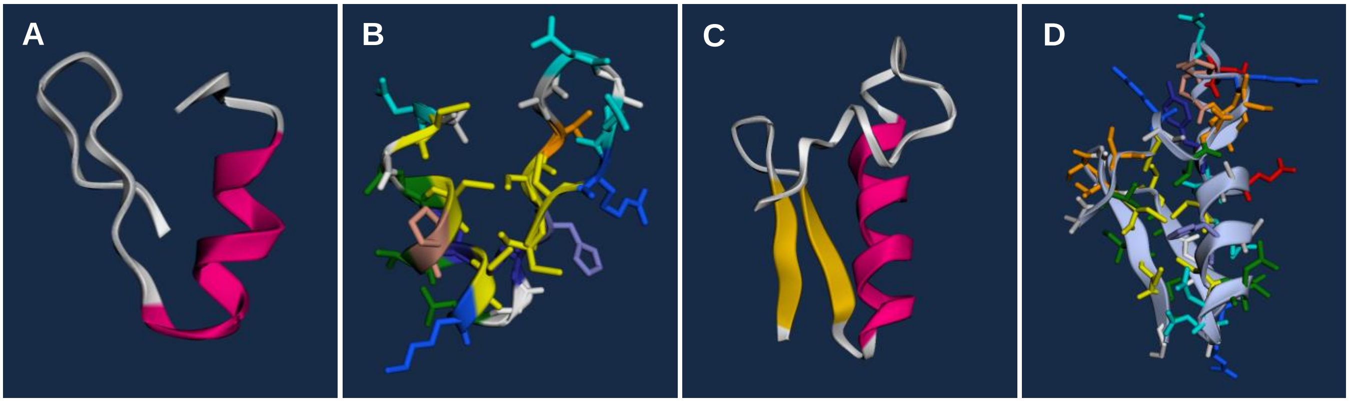


**Supplementary Material S4**. Prediction metrics of AlphaFold 2 (A) and distance calculation (B) performed for sulfur atoms of the Cysteine based on the three-dimensional model from AlphaFold 2.
